# Does the mode of delivery in Cardiac Rehabilitation determine the extent of psychosocial health outcomes?

**DOI:** 10.1016/j.ijcard.2017.11.056

**Published:** 2018-03-15

**Authors:** Alex S. Harrison, Patrick Doherty

**Affiliations:** Department of Health Sciences, University of York, York, UK

**Keywords:** Exercise, Rehabilitation, Quality of life, Anxiety, Depression, Secondary prevention

## Abstract

**Background:**

Cardiac Rehabilitation (CR) is a multicomponent tailored intervention aiming to reduce lifestyle risk factors and promote health in patients post cardiovascular disease. CR is delivered either as supervised or facilitated self-delivered yet little evidence exists evaluating the association between mode of delivery and outcomes.

**Methods:**

This observational study used data routinely collected from the National Audit of Cardiac Rehabilitation from April 2012–March 2016. The analysis compared the populations receiving supervised and facilitated self-delivered modes for differences in baseline demographics, four psychosocial health measures pre and post CR and changes in anxiety, depression and quality of life following the intervention. The analysis also modelled the relationship between mode and outcomes, accounting for covariates such as age, gender, duration and staffing.

**Results:**

The study contained 120,927 patients (age 65, 26.5 female) with 82.2% supervised and 17.8% self-delivered. The analysis showed greater proportion of females, employed and older patients in the self-delivered group. Following CR, patients in both groups demonstrated positive changes which were of comparable size. The regression model showed no significant association between mode of delivery and outcome in all four psychosocial outcomes when accounting for covariates (*p*-value > 0.0.5).

**Conclusions:**

Patients benefited from attending both modes of CR showing improved psychosocial health outcomes with 3–76% change from baseline. Over half of CR programmes in the UK do not provide self-delivered CR yet this mode is known to reach older patients, female and employed patients. Facilitated self-delivered CR should be offered and supported as a genuine option, alongside supervised CR, by clinical teams.

## Highlights

• Self-delivered population is older and greater proportion female.
• Patients have psychosocial benefit from attending either supervised or self-delivered.
• UK National audit concludes in representative routine population self-delivered.
• Supervised and self-delivered rehabilitation patients have similar psychosocial health outcomes.

## 1 Introduction

Cardiac Rehabilitation (CR) is a strongly evidenced intervention that is recognised as integral to comprehensive care for a range of cardiac conditions and treatments [1], [2], [3]. CR had, in 2007, a class one recommendation from the American Heart Association, American College of Cardiology and the European Society of Cardiology in the care of patients with heart disease [1], [4].

The evidence for CR can be split into trial evidence and modern observational clinical registries [1], [2]. The trial data, for the effectiveness of CR, summarised by the most recent Cochrane review shows that CR reduces cardiovascular mortality (RR 0.74, 95% CI 0.64–0.86) and hospital re-admissions post CR (RR 0.82, 95% CI 0.70–0.96) [1]. The registry data shows that CR could also significantly reduce all-cause mortality (HR 0.37, 95% CI 0.20–0.69) [3]. This disparity in conclusions highlights the differing populations that the studies/trials incorporate. In that Cochrane review average patient age was 56 years, whereas in the 2016 National Audit of Cardiac Rehabilitation (NACR) patients in the UK were shown to be 65 years, a 9 year increase in average age [1], [5]. This issue of representativeness is a justification for increased use of observational registry based research.

Currently, the UK is world leading with 50% uptake across the four main diagnosis/treatment groups, Myocardial Infarction (MI), Percutaneous Coronary Intervention (PCI), MI + PCI, and Coronary Bypass Graft (CABG) [5]. Modern CR remains dominated by group-based approaches, with 82% of all patients taking up this mode of delivery as evidenced through the NACR 2016 report [5]. In 2017 a review concluded, based on 23 trials, that home based versus centre based rehabilitation was not associated with patients' outcomes, including physical capacity, mortality and health related quality of life. This strongly supports the utilisation of a diverse menu based approach to CR, which would include group based, home based and manual based CR [6]. However, in 2016 only ~ 60% of programmes in the UK did not have patients receiving home-based in the 2016 audit [5]. Additionally, as shown in the review of CR effectiveness, evidence based on trial populations is often not representative of routine care. In the home vs. centre review 6 trials contained no female participants, when routine care shows around 30% female participation [1], [6].

The traditional mode of CR delivery in Europe is supervised CR, with a median of 12 months with exercise as a predominant factor [1], [2], [5], [7], [8]. Alternatively, facilitated self-delivered structured programmes such as the Heart Manual, Angina plan and home-based CR exist which are completed over a similar period [5], [6], [7], [8]. The two forms of delivery, supervised versus facilitated self-delivered CR, are now forming modern CR. There is debate whether supervised delivery is better than its structured self-delivered counterpart containing facilitation from the CR team, as described in the heart manual [8]. A Danish study, from the CopenHeart research group, allocated patients into supervised group-based or self-care home-based; the findings were similar to that of the Cochrane Review and trial in favour of equivalence [9].

The British Association for Cardiovascular Prevention and Rehabilitation (BACPR) core components state that CR can be delivered in a variety of ways such as centre based and home-based along with the trial evidence that exists to suggest a comparable association with outcomes [10]. This study aims to investigate whether in a routine care population there is an association between patients receiving supervised or self-delivered CR and their psychosocial health outcomes post-CR. This will build upon the trial evidence, but in a more representative and diverse population.

## 2 Methods

This study was reported according to the Strengthening the Reporting of Observational Studies in Epidemiology (STROBE) guidelines [11].

### 2.1 Data

The planned analyses used routinely collected patient-level data from the UK NACR database from 1st April 2012 to 31st March 2016. NACR collects electronic patient-level data from over 226 programmes each year [5].

NACR collects information about patients going through CR such as initiating event, treatment type, individual risk factors, medication use, patient characteristics and outcomes, along with centre level information; volume and staffing profiles [5]. Data is collected under NHS data requirements, reviewed annually by NHS Digital, which hosts and oversees the quality of audit data in the NHS. All data used in this study is anonymised by NHS Digital before reaching the NACR team.

CR is recommended for patients with a diagnosis of MI, heart failure, and angina; along with being eligible after having a treatment of CABG, PCI and Pacemaker [12], [13], [14]. All patients entered into the audit, within the time period, with an in scope diagnosis or treatment were included in the analysis [5].

The study includes CR programmes in the UK, with valid patient data at both pre and post CR assessment and completed data fields capturing staffing information. Inclusion was based on all patients with a valid diagnosis/treatment, started CR and a mode of delivery completed; this population was verified against the whole CR population without these measures completed (matching age, gender and baseline scores).

### 2.2 CR/Mode of delivery

Nationally CR is expected to be conducted according to the BACPR core components, which recommends a patient-tailored approach, based on the baseline assessment, defined needs and patient preference [10]. Patient specific CR means that mode of delivery is a patient-level variable, whereas staffing type is programme level.

For this study mode of delivery was coded from NACR variables, including group-based, home-based and web-based, into supervised (with staff present) and facilitated self-delivered (with contact but no staff required for the exercise component). Patients recorded as receiving delivery classified as ‘other’ were excluded from the study due to lack of descriptive information; this equalled 3% of patients, and were assessed for differences in demographics to ensure our final sample was representative.

### 2.3 Outcome measures

Psychosocial health status is a core area for CR, which in the UK includes assessment of the extent of anxiety, depression, self-perceived feelings and Quality of Life (QoL) at baseline and following CR as a measure of outcome improvement. Before starting, the 8–12 week CR programme all patients should receive a baseline assessment, which includes the Hospital Anxiety and Depression Scale (HADS) and Dartmouth questionnaire. This records their psychosocial well-being at baseline, which helps tailor the intervention. The patient is then provided a follow-up assessment post CR that assesses their improvement across the intervention. The outcomes included were HADS for anxiety and depression and the Dartmouth questions for Quality of Life (QoL) and feelings. HADS Anxiety and depression symptoms were separately measured (score range 0–21) with higher scores representing worse symptoms; patients were grouped by score as normal category (≤ 8) and at-risk group (8 +) [15], [16]. The Dartmouth feelings and QoL questions provide self-perceived psychosocial health scores. Responses were coded 1–5 and were dichotomised (normal score 1–3, at-risk score 4–5) [11].

### 2.4 Statistical analysis

The analyses were conducted in STATA 13.1. Baseline characteristics were compared across groups using Chi^2^ and odds ratios for categorical variables or *t*-test for continuous variables. Regression models were built to investigate whether, accounting for covariates, the supervised and self-delivered methods for mode of delivery were associated with outcomes post CR.

Relevant important covariates were included in the analysis, where they were evidenced in the literature or significant in preliminary analysis. Age (years), gender (male/female), number of comorbidities and employment status have been shown to influence the outcomes following a variety of different interventions, including CR [16], [17], [18]. Employment status was coded as employed/retired or unemployed, this is because previous research found that employed and retired states have similar effects on outcomes [16]. The duration of CR (length of CR) was also included in the analysis along with staffing profile, total staff hours, Multi-Disciplinary Team (MDT) and total centre volume. The staffing information comes from the annual survey, performed routinely by the NACR to gain centre level information such as staff profile, hours and funding type. Because the mode of delivery was a patient-level variable, it was important to take into account the relative size and staffing profile of the centre where the patient received the CR.

Hierarchical logistic regressions were used to investigate the association between mode of delivery, as an independent variable, and psychosocial health outcomes as the dependent variable. A hierarchical design was used to account for different levels of patient and centre level data. Statistical level for significance was *p* < 0.05. Data model checking was performed to ensure that the models were a good fit through assumptions associated with the regressions.

## 3 Results

### 3.1 Study population

**Fig. 1:**
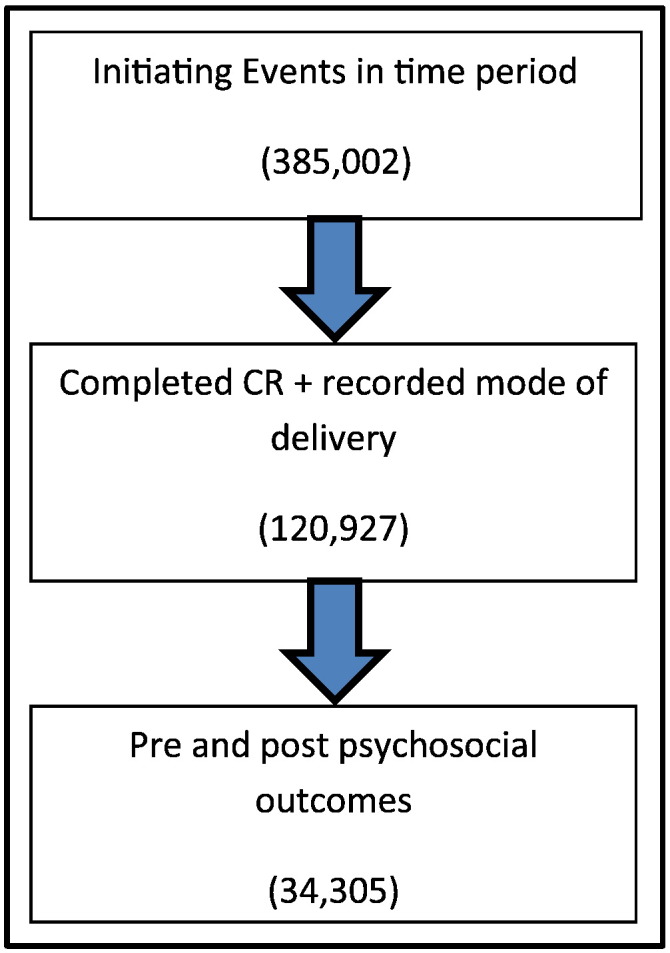
Flow diagram showing total population in time period, those with valid mode of delivery and those with pre and post outcome measures resulting in them being included in regression analysis

The study included 120,927 valid cases from across the UK that attended CR in the four-year period, this was from a sample of 385,002 patients entered in the time period, shown in Fig. 1. Within our eligible population, 82.3% received supervised CR whereas 17.7% received CR such as home-based or web-based coded as self-delivered.

The analysis in Table 1 shows increased odds for females and employed patients receiving self-delivered CR (1.26 and 1.24). The analysis also showed that older patients, lower mean comorbidity and longer duration were significantly associated with patients receiving self-delivered CR.

**Table 1:** Showing the differences at baseline of patients when split by the mode of delivery they receive at CR.

	Supervised	Self-delivered	Total	Mean difference/Pearson Chi-square value	Odds ratio (CI 95%)
Number of patients (%)	99,491 (82.3%)	21,436 (17.7%)	120,927 (100.0%)		
Female (%)	25,190 (25.6%)	6565 (30.8%)	31,755 (26.5%)	258.356 (< 0.001)	1.29 (1.26–1.32)
Mean age (SD)	64 (12)	67 (12)	65 (12)	2.282 (< 0.001)	
Mean number of comorbidities (SD)	1.6 (1.6)	1.5 (1.5)	1.6 (1.6)	0.13089 (< 0.001)	
Mean duration of CR days (SD)	68 (42.9)	87 (61.9)	69 (47.3)	19.310 (< 0.001)	
Employed count (%)	42,012 (70.5%)	7940 (74.8%)	49,952 (71.2%)	143.29 (< 0.001)	1.24 (1.19–1.28)

Table 2 shows the baseline scores for psychosocial health measures across the two different modes of delivery. The Chi^2^ analysis shows that there is significant difference between the two groups. The estimated odds ratio shows the size of the difference, which is 9–27% less likely to be in the target normal group at baseline if the patient attends self-delivered CR. This suggests that patients with poorer psychosocial health at baseline are receiving self-delivered CR compared to the supervised mode of delivery population. The patients are on average more anxious, depressed or have poorer psychosocial health in the self-delivered group.

**Table 2:** The differences in percentage of patients in normal group at baseline and change post CR for the four outcome measures, HADS Anxiety and Depression, Dartmouth Feelings and Quality of Life.

	% in normal category for psychosocial health state			Odds ratio (Chi^2^*p*-value)	% Change into normal category by mode of delivery type the patient received		
	Supervised	Self-delivered	Total		Supervised	Self-delivered	Total
HADS Anxiety percentage in normal group	72.0%	70.2%	71.8%	0.91 (0.005)	6	7	6
HADS Depression percentage in normal group	82.1%	79.3%	81.8%	0.84 (< 0.001)	6	7	6
Dartmouth Feelings percentage in normal group	84.9%	83.2%	84.7%	0.88 (0.004)	5	6	5
Dartmouth Quality of Life percentage in normal group	95.2%	93.5%	95.0%	0.73 (< 0.001)	3	4	3

The percentage change in Table 2 shows that all patients, in either delivery group, benefit from CR and demonstrated positive change. The self-delivered group actually improves more across all four outcomes measures, however, as seen in Table 2 this group also starts at a lower percentage at baseline.

### 3.2 Outcomes

Table 3 shows the results from the Logistic regression, comparing supervised delivery to self-delivered. In total 34,000 were eligible for the analysis with pre and post psychosocial measures recorded as shown in Fig. 1. The numbers included in each model are presented in Table 3. There was no significant association seen between any of the outcomes and the mode of delivery. The covariates that were included were justified. Employment status, age, sex and comorbidities, staffing hours and MDT were all seen to be significantly associated with likelihood of achieving the target health state post CR. All assumptions for the type of model used were met.

**Table 3:** Results from the hierarchical logistic regression analysis; association between mode of delivery and psychosocial health outcomes post CR.

Effect of self-delivered in comparison to supervised					
	Odds ratio	Sig.	95% CI		Observations
HADS Anxiety	0.981	0.744	0.878	1.098	33,748
HADS Depression	1.099	0.158	0.964	1.254	33.719
Dartmouth Feelings	0.882	0.086	0.764	1.018	28,982
Dartmouth Quality of Life	0.830	0.134	0.650	1.059	28,982

## 4 Discussion

The results from this study show that patients benefit irrespective of the mode of delivery in terms of psychosocial health outcomes following CR. This is the first large-scale routine population study to investigate whether the type of delivery influences the outcomes in a routine clinical setting. This study builds on the trial conclusions from Cochrane review by identifying in a real world setting that there is no significant association between different CR types and psychosocial health outcomes [6]. The results from the regression, that mode of delivery that a patient receives does not have an association with post CR psychosocial health outcome, is likely because CR is structured and patient-tailored, thus following the structure results in positive change.

The study's population consisted of 120,927 patients that were representative of modern UK CR. The population included in the valid case analysis was checked against the non-valid population; the valid population was deemed not significantly different in age, gender and baseline psychosocial health measures. The age, gender and comorbidity demographics were similar to the 2016 annual report [5]. However, the demographic profile shows stark contrast to the findings of the two recent Cochrane reviews which showed 15% female participation, as opposed to our ~ 26%, and 56 mean age where as this study had 65 (SD 12) [1], [6]. This shows the difficulty between using trial evidence and the routine populations for generating service level advice. The recent CROS review, that utilised registry data from Europe, shows a similar population to this study, which supports the differences in routine clinical populations and those seen in trials [2].

The analysis investigated whether the patients receiving the two types of delivery differed at baseline; it showed that older, employed and female patients tended to be within the self-delivered programme. This is extremely important because female and older patients are often deemed in the evidence to be hard to reach and not taking up the offer of CR. If there is a preference in these demographics for self-delivered CR then a more diverse menu based approach to CR could influence uptake.

Patients were also investigated for differences pre and post CR in terms of psychosocial health. It was shown that patients in the self-delivered group were less likely to be in the normal group at baseline (0.91–0.73), however, they experienced a greater change post. This supports the idea that those with the most to gain experience the highest change and the supervised group was experiencing a ceiling effect. Regardless of this difference in change, the regression model shows no association between mode of delivery and post CR score.

This study's results emphasise the trend seen in recent literature that mode of delivery defined as supervised or self-delivered does not alter patient's outcomes. In the UK only 40% of centres supported patients receiving self-delivered CR which shows a lack of diversity in delivery [5]. This study shows that older and female patients may be more likely to attend self-delivered CR. The 65% uptake ambition set by NHS England [19] and 70% from the recent Road Map for CR [20], remains challenging and can only be achieved if CR programmes offer a greater choice to patients by offering more diverse CR options.

The regression analyses showed that there was no difference in psychosocial health outcomes post CR between the modes of delivery. There was positive change gained regardless of mode of delivery, which shows that both methods of CR lead to improvements in psychosocial health.

In 2016, the NACR reports 50% uptake with 82% receiving supervised group-based CR, perhaps to further increase uptake the numbers receiving self-delivered programmes should increase [5]. The remaining patients not taking up the offer of CR, due to the offer not appealing, are branded harder to reach and are often female and older. This study suggests that the composition of facilitated self-delivery contained older patients with a higher proportion of females; this suggests that higher utilisation of this mode of delivery will improve the offer of CR and thus improve uptake.

### 4.1 Limitations

Our study population had a good size and is considered representative of modern routine CR. The study results, which reflect routine clinical practice, build on what was found in the Cochrane review of clinical trials in that mode of delivery is not a determinate of outcomes and that providing high quality tailored CR is associated with improved outcomes regardless of mode of delivery [6].

This study used four years of NACR accumulated data, which after including all the different variables such as age, gender, comorbidities and mode of delivery amounted to 120,927 patients. One limitation that is shared with the NACR 2016 national report is that only 56% of patients that start CR have a recorded post assessment. This reduces the number of valid patients substantially for the later analysis. The population is still representative and the analysis has enough patients. However, improvements in the recording of data such as mode of delivery, post assessments and baseline demographics would improve the power given to research such as this.

In addition to completeness of data, there are some issues around the use of questionnaires to capture patients' psychosocial health, firstly collecting questionnaires post an intervention may reduce completeness and secondly, honesty of patients recording psychosocial health may be questioned. These two issues could lead to recall and collection bias, however, the two questionnaires were validated in our CVD population and the authors feel confident of the accuracy of the outcomes.

Another limitation of this study is the level of contact that the CR team had with the self-delivered programme. The self-delivered programme was defined from modes such as home-based and web-based which are structured programmes facilitated by the CR, the exact nature of the facilitation specific to programmes was unknown.

## 5 Conclusion

This is the first investigation of the association between mode of delivery and psychosocial health outcomes in the UK clinical setting. This study aimed to investigate whether supervised or self-delivered CR differed in terms of four psychosocial health outcomes. This study concluded that there is no association between mode of delivery and psychosocial health outcomes post-CR. Currently, in the UK there are ~ 60% of programmes not providing self-delivered CR, with this study and the growing evidence there should be a wider menu of options in the delivery of CR including facilitated self-delivered programmes. This study suggests that facilitated self-delivered CR is appealing for older, female and employed patients who are traditionally harder to reach, through wider implementation of self-delivered uptake which may increase further from 2016.

## Funding

This research was carried out by the British Heart Foundation (BHF) Cardiovascular Health Research Group which is supported by a grant from the BHF (R1680901).

## Conflict of interest

None.
